# Therapeutic management of PI3Kα inhibitor-induced hyperglycemia with a novel glucokinase activator: Advancing the Frontier of PI3Kα inhibitor therapy

**DOI:** 10.1016/j.molmet.2025.102151

**Published:** 2025-04-14

**Authors:** Guanqin Jin, Shihuang Liu, Kewei Zheng, Xiaobo Cheng, Ranran Chai, Wei Ye, Wei Wei, Yongguo Li, Ai Huang, Guiling Li, Huan Yi, Yu Kang

**Affiliations:** 1 Clinical Research Center, Obstetrics and Gynecology Hospital, Fudan University, Shanghai, 200011, China; 2 Shanghai Key Laboratory of Female Reproductive Endocrine Related Diseases, Fudan University, Shanghai, 200011, China; 3 Department of Obstetrics and Gynecology, Obstetrics and Gynecology Hospital, Fudan University, Shanghai, 200011, China; 4 Department of Gynecologic Oncology, Fujian Maternity and Child Health Hospital College of Clinical Medicine for Obstetrics & Gynecology and Pediatrics, Fujian Medical University, Fuzhou, 350001, China; 5 Fujian Province Key Clinical Specialty for Gynecology, Fujian Key Laboratory of Women and Children's Critical Diseases Research, National Key Gynecology Clinical Specialty Construction Institution of China, Fuzhou, 350001, China; 6 Department of translational medicine, Shanghai Jiatan Pharmatech CO. LTD, Shanghai, 201203, China; 7 Cancer Center, Union Hospital, Tongji Medical College, Huazhong University of Science and Technology, Wuhan, 430022, China; 8 Hubei Key Laboratory of Precision Radiation Oncology, Wuhan, 430022, China

**Keywords:** PI3K inhibitor (PI3Ki), Hyperglycemia, Insulin signaling pathway, AKT/INSR/mTOR, Dorzagliatin, Blood glucose management, HK-2

## Abstract

**Objectives:**

The phosphatidylinositol 3-kinase (PI3K) signaling pathway is a pivotal target in cancer treatment, driving substantial investigation into PI3K inhibitors (PI3Ki). However, the common on-target adverse effect of hyperglycemia presents a substantial challenge to their clinical application. There is an urgent need to discover an anti-hyperglycemic agent that maintains the efficacy of PI3Ki.

**Methods:**

We conducted a comprehensive study to explore the interaction between exogenous hyperinsulinemia and PI3Ki in SKOV3 and OVCAR3 ovarian cancer cell lines. We used Western blotting, CCK-8, and EdU assays to determine the effect of this interaction on cell proliferation. In addition, we evaluated the anti-hyperglycemic effects of dorzagliatin in a PI3Ki-induced hyperglycemic mice model. Cell line-derived xenograft (CDX) models were employed to evaluate the in vivo tumor growth inhibitory effects of combining dorzagliatin with PI3Ki.

**Results:**

Western blot analysis demonstrated that insulin activated the AKT/INSR/mTOR pathway, reversing PI3Ki-induced p-AKT inhibition. Insulin also attenuated the anti-proliferative effects of PI3Ki. In the hyperglycemic mouse model, dorzagliatin significantly reduced blood glucose levels compared to controls. The combination therapy group (Dorzagliatin + PI3Ki) in CDX models showed a marked reduction in tumor volume. Dorzagliatin not only mitigated hyperglycemia but also enhanced the anti-tumor effects of PI3Ki. A clinical trial (NCT06117566) in cervical cancer patients supported these findings, showing that dorzagliatin stabilized blood glucose levels, facilitated body weight recovery, and achieved a confirmed partial response (PR).

**Conclusions:**

Dorzagliatin shows promise for managing PI3Ki-associated hyperglycemia, thereby enhancing its therapeutic efficacy. The activation of liver glycogen kinase and insulin regulation may be key mechanisms underlying its therapeutic benefits.

## Highlights

• Insulin has been shown to diminish the antitumor activity of PI3Ki in SKOV3 and OVCAR3 cell lines.
• Dorzagliatin is capable of reducing PI3Ki-induced hyperglycemia and hyperinsulinaemia in BALB/c mice. It significantly augments the anticancer effects of PI3Ki in CDX models and attenuates HK-2 activity.
• A clinical case further corroborates that dorzagliatin can mitigate PI3Ki-induced hyperglycemia and ultimately lead to a confirmed partial response.

## 1 Introduction

The phosphatidylinositol 3-kinase pathway is critical for cellular metabolism and growth regulation. PIK3CA mutations, which lead to constitutive activation of PI3Kα, are frequently observed across multiple cancer types, particularly in breast, endometrial, and ovarian malignancies [1,2]. The development of PI3Kα inhibitors has emerged as a promising therapeutic strategy, demonstrating remarkable efficacy in both preclinical models and clinical trials for PIK3CA-mutated solid tumors[[3], [4], [5], [6]]. Alpelisib, a selective PI3Kα inhibitor, has received regulatory approval for treating hormone receptor-positive (HR+), HER2-negative breast cancer harboring PIK3CA mutations [7]. Clinical trials have shown alpelisib to be a positive treatment option for patients with recurrent gynecologic cancer and a PIK3CA mutation [8]. In addition to antitumor therapy, the Food and Drug Administration (FDA) has given rapid approval of alpelisib for the treatment of adults and children 2 years of age or older who have severe manifestations of PIK3CA-related overgrowth spectrum (PROS) and need systemic medication [9].

However, the clinical utility of PI3Kα inhibitors is significantly limited by their metabolic adverse effects, particularly hyperglycaemia [5,10,11]. PI3Kα plays a physiological role in insulin signaling and glucose homeostasis, contributing to this on-target impact. In the SOLAR-1 trial, 63.7% of alpelisib-treated patients experienced hyperglycemia of any grade and 36.6% experienced grade 3–4 hyperglycemia. The alpelisib discontinuation rate due to hyperglycemia was 6.3% [12]. WX390 is a highly potent PI3K-mTOR dual inhibitor with high potency to PI3Kα [13]. In a Phase Ib/II clinical trial assessing the combination therapy of WX390 and Toripalimab, hyperglycemia was reported as a treatment-related adverse event (TRAE) in 75.4% of the participants. Notably, 35.4% of these patients experienced hyperglycemia at a severity level of Grade ≥3 [14]. More concerning is the observation that treatment-induced hyperglycaemia triggers compensatory insulin secretion, which can potentially reactivate the PI3K/AKT pathway in tumor cells, leading to therapeutic resistance [15].

Current strategies for managing PI3Kα inhibitor-induced hyperglycaemia present significant limitations. Exogenous insulin administration, while effective in controlling hyperglycaemia, may paradoxically compromise antitumor efficacy [16]. Traditional insulin-sensitising agents share similar drawbacks, while ketogenic diets might stimulate tumor growth through PPAR pathway activation [17]. Although pioglitazone shows promise in preventing PI3K inhibition-associated hyperglycaemia without affecting antitumor activity [18], its delayed onset of action limits its utility as a first-line intervention [19]. Sodium-glucose cotransporter-2 inhibitors (SGLT2i), while efficient in decreasing blood glucose without raising insulin levels, involve hazards of ketoacidosis and unintentional weight loss [20,21].

In this context, dorzagliatin, a novel glucokinase activator, emerges as a potentially valuable therapeutic option [22,23] by restoring glucose homestasis and promoting glycogen synthesis [24]. Additionally, it modulates pancreatic β-cell function, enhancing early-phase insulin secretion to quickly regulate blood glucose levels. This dual mechanism stabilizes blood glucose while minimizing systemic insulin impact [25], making dorzagliatin an attractive candidate for managing PI3K inhibitor-induced hyperglycemia and potentially maintaining PI3K inhibitor efficacy without the insulin feedback associated with other antidiabetic therapies.

This study aims to evaluate dorzagliatin's ability to regulate PI3K inhibitor-induced hyperglycaemia and its impact on the antitumor efficacy of these agents in PIK3CA-mutant ovarian cancer. We hypothesise that dorzagliatin's glucose-lowering effect, achieved without significant hyperinsulinaemia, could maintain the therapeutic benefits of PI3K inhibition while mitigating its metabolic complications.

## 2 Material and methods

### 2.1 Mouse experiments

All animal-related operations were carried out in compliance with protocols authorized by WuXi AppTec's Institutional Animal Care and Use Committee (IACUC), which followed the Guide for the Care and Use of Laboratory Animals, Eighth Edition (2011). During routine monitoring, the animals were examined daily for any effects of tumor growth and treatments on normal behavior, such as mobility, food and water consumption, body weight (measured three times per week), and visual signs of adverse effects, such as eye/hair matting or other abnormalities. Mortality and clinical symptoms were collected for each experimental group.

Eight-week-old BALB/c mice were acquired from Charles River (Zhejiang, China) and acclimated for at least one week with free access to water and food prior to treatment. Mice were assigned at random to six treatment groups and treated daily with oral gavage for 4 weeks as follows: (1) vehicle control [1% wt/vol.] methylcellulose in water, *n*=6); (2) dorzagliatin (10 mg/kg, *n* = 6); (3) WX390 (0.2 mg/kg, *n* = 6), (4) BYL719 (25 mg/kg, *n* = 6), (5) WX390 (0.2 mg/kg, *n* = 6) + dorzagliatin (10 mg/kg, *n* = 6); and (6) BYL719 (25 mg/kg, *n* = 6) + dorzagliatin (10 mg/kg, *n* = 6). Dorzagliatin was administered 30 min prior to PI3Ki to synchronize peak blood glucose levels with its optimal efficacy.

For blood glucose level studies, blood samples were collected at 1, 2, 4 and 8 h after treatment. Body weight changes and mortality were recorded 2 days after treatment initiation. For antitumor efficacy studies, BALB/c nude mice were injected under the skin with 0.5–1 × 10^6^ cells in a 1:1 blend of growth medium and Matrigel (356234, Corning, NY, USA). Tumors were allowed to grow to a minimum diameter of 0.6 cm before treatment began. At the end of the trial, blood was drawn 1, 2, 4, and 8 h after treatment, and the animals were euthanized. Three mice from each group were randomly selected to collect tumor and liver samples 2 h after administration, which were then frozen in liquid nitrogen and stored at −80 °C for subsequent analysis.

### 2.2 Chemicals

Human insulin (1 mg/ml in hydrochloric acid, pH 2–3) was provided from Absin (Shanghai, China). BYL719 was supplied by Selleckchem (Houston, TX, USA), and dissolved in DMSO at 10 mM. Dorzagliatin and WX390 were provided by Joyo Pharmatech (Shanghai, China).

### 2.3 Cell culture and cell proliferation assay

Ovarian cancer cell lines SKOV3 and OVCAR3 were obtained from the Shanghai Key Laboratory of Female Reproductive Endocrine Related Diseases, Obstetrics and Gynecology Hospital, Fudan University, Shanghai, China. Cells were cultured in RPMI-1640 medium supplemented with 10% fetal bovine serum (FBS; BasalMedia, Shanghai, China) and 1% Penicillin-Streptomycin (NCM Biotech, Suzhou, China) under standard conditions (37 °C, 5% CO_2_). Cell proliferation was evaluated using the CCK-8 Kit (NCM Biotech, Suzhou, China), adhering to the manufacturer's guidelines. Cells, seeded at 1 × 10^3^ cells/well in 96-well plates, were treated with BYL719 (5 μM)/WX390 (1 μM) alone or in combination with insulin (10 ng/ml) for 1–4 days and then incubated with CCK-8 solution for 2 h. Absorbance was subsequently measured at 450 nm using a Multimode Plate Reader (Molecular Devices, USA).

### 2.4 EdU staining assays

Cell proliferation was analyzed using EdU staining according to the following protocol. Cell lines were treated with BYL719 (5 μM)/WX390 (1 μM) alone or in combination with insulin (10 ng/ml) for 48 h in six-well plates and tested using an EDU test kit (Beyotime, Shanghai, China). The EdU incorporation assays were conducted as per the manufacturer's guidelines. Nuclei were counterstained with 4′,6-diamidino-2-phenylindole (DAPI; C0060-1 ml, Solarbio, Beijing, China). The results were observed under fluorescence microscopy and the EDU positive cells in different treatment groups were analyzed.

### 2.5 Western blotting (WB)

Cells were exposed to BYL719 (12 μM) alone for 1 h or in combination with insulin (10 ng/ml) for 10 min before harvesting. Cell lysates were prepared using in RIPA buffer containing protease and phosphatase inhibitors (Beyotime, Shanghai, China). Tissue specimens intended for WB analysis were first excised and immediately placed on ice. The tissues were weighed and then homogenized in ice-cold RIPA buffer supplemented with protease and phosphatase inhibitors (Beyotime, Shanghai, China) using a mechanical homogenizer. The homogenates were centrifuged at 12,000×*g* for 10 min at 4 °C to remove cellular debris, and the supernatants were collected as tissue protein lysates. Protein concentrations were quantified via a BCA Protein Assay Kit (Beyotime, Shanghai, China). Equivalent protein aliquots were separated on 4–20% Tris-Glycine gels (Thermo Fisher, Carlsbad, CA, USA) and subsequently transferred to PVDF membranes. Membranes were probed overnight at 4 °C with primary antibodies against pAKT473 (Cell Signaling Technology Cat# 4060, RRID:AB_2315049, 1:1000 dilution), pAKT308 (Cell Signaling Technology Cat# 13038, RRID:AB_2629447, 1:500 dilution), INSR (Cell Signaling Technology Cat# 3025, RRID:AB_2280448, 1:500 dilution), pINSR (Cell Signaling Technology Cat# 3021, RRID:AB_331578, 1:500 dilution), pmTOR (Cell Signaling Technology Cat# 5536, RRID:AB_10691552, 1:500 dilution), mTOR (Thermo Fisher Scientific Cat# PA5-17780, RRID:AB_10979187, 1:500 dilution), AKT (Starter Biotechnology Cat# S0B0114, 1:500 dilution), and GAPDH (Starter Biotechnology Cat# S0B0261, 1:20000 dilution). Following three washes with TBST, membranes were incubated with secondary antibodies at room temperature for 1 h and visualized using enhanced chemiluminescence (NCM Biotech, Suzhou, China).

### 2.6 Blood glucose and C-peptide measurements

Blood glucose was quantified with a OneTouch Ultra Glucometer. Blood samples (10 μl) were drawn from the tail vein of the mice at baseline (time 0) and at 0.5, 1, 2, 4, 6, and 8 h following the administration of treatment. For plasma collection, blood (≥100 μl) was collected into EDTA tubes (16.444, Sarstedt, Nümbrecht, Germany), centrifuged at 10,000×*g* for duration 10 min at 4 °C, and stored at −20 °C. Plasma C-peptide concentrations were measured using an ELISA kit (90050, Crystal Chem, IL, USA).

### 2.7 Statistical analysis

Data are presented as means ± standard error of measurement (SEM). Statistical analyses were conducted using GraphPad Prism version 10.1.0 (GraphPad, La Jolla, USA). Group differences were analyzed using Student's t-test, unpaired t-test, or one-way ANOVA, as appropriate. Statistical significance was defined as p < 0.05. Symbols for significance were as follows: ∗ for p < 0.05, ∗∗ for p < 0.01, ∗∗∗ for p < 0.001, and ∗∗∗∗ for p < 0.0001.

## 3 Results

### 3.1 Exogenous hyperinsulinism reduces the antitumor activity of PI3Ki

**Figure 1:**
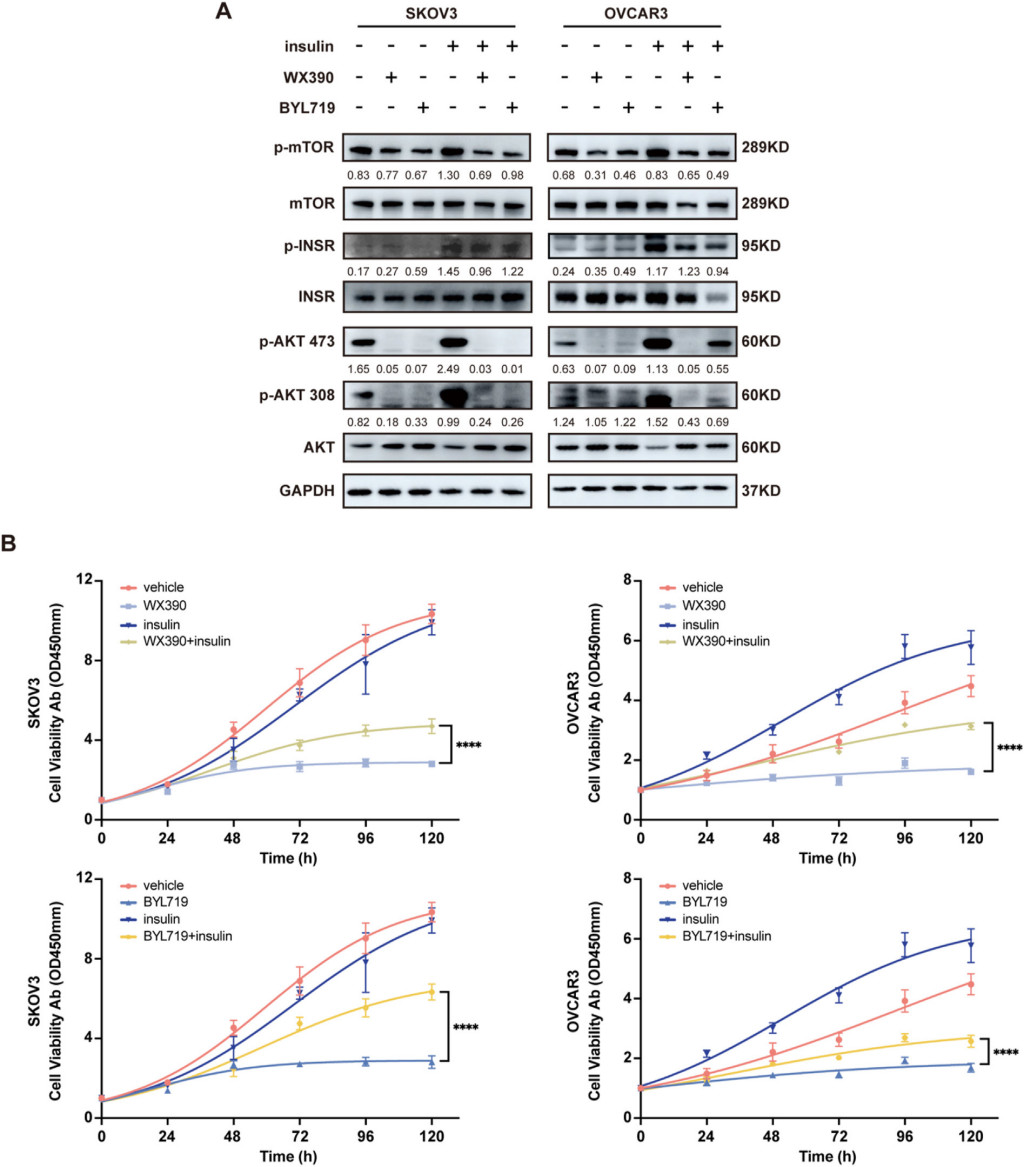
**Exogenous hyperinsulinism reduces the antitumor activity of PI3Ki.** (A) Western blot analysis showing changes in the PI3K-mTOR signalling pathway after treatment with insulin and PI3Ki (WX390 or BYL719) in SKOV3 and OVCAR3 cell lines. (B) Proliferative capacity of tumor cells measured by CCK-8 assay following treatment with insulin and PI3Ki. Statistical significance was determined by one-way ANOVA: ∗∗∗∗p < 0.0001.

Clinically, PI3Ki have been found to cause insulin resistance, leading to hyperglycemia and compensatory hyperinsulinaemia [5]. We hypothesised that PI3Ki-induced hyperinsulinaemia may counteract their antitumor effects by reactivating cell proliferation through the AKT/INSR/mTOR signalling pathway. Western blot analyses confirmed this hypothesis, showing that insulin treatment markedly increased the phosphorylation of AKT, INSR, and mTOR in tumor cells (Figure 1A). Furthermore, the inhibitory effect of BYL719 on AKT phosphorylation (p-AKT) was reversed in the presence of insulin, indicating pathway reactivation. Notably, while insulin reversed the inhibitory effect of BYL719 on p-AKT in the OVCAR3 cell line, this reversal was not observed in SKOV3 cells.

**Figure 2:**
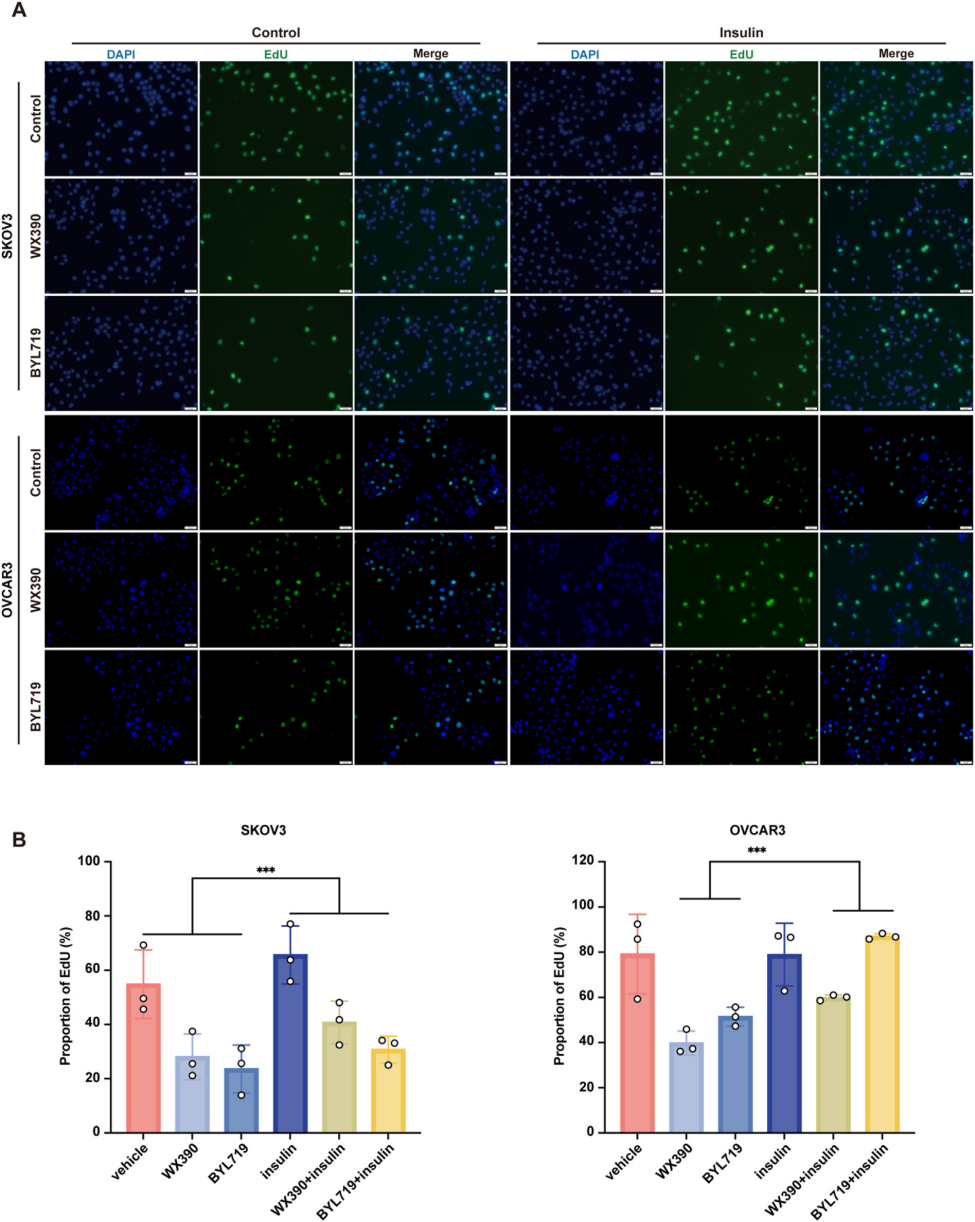
**Exogenous hyperinsulinism impaired the antiproliferative effects of PI3Ki.** (A–B) EdU assay results showing changes in tumor cell proliferation in SKOV3 and OVCAR3 cell lines after treatment with insulin and PI3Ki. Data are presented as mean ± SD; statistical significance was determined by one-way ANOVA: ∗∗∗p < 0.001.

To further investigate the effect of hyperinsulinaemia on the efficacy of PI3Ki, we used CCK-8 and EdU cell proliferation assays on SKOV3 and OVCAR3 ovarian cancer cell lines treated with insulin (10 ng/ml), WX390 (1 μM) and BYL719 (5 μM). Results demonstrated that elevated insulin levels significantly impaired the antiproliferative effects of WX390 and BYL719, as insulin-treated tumor cells exhibited increased proliferation compared to those treated with BYL719 alone (Figure 1B). Notably, in the EdU assay, while SKOV3 cells exhibited a significant proliferative increase with insulin in both the vehicle- and PI3K inhibitor-treated conditions, a statistically significant increase in proliferation was observed in OVCAR3 cells only in the PI3K inhibitor-treated group, with no significant change in the vehicle-treated cells (Figure 2A–B). These findings suggest that hyperinsulinaemia, often secondary to PI3Ki-induced hyperglycaemia, may compromise the therapeutic effect of PI3Ki by promoting tumor cell proliferation.

### 3.2 Dorzagliatin reduces PI3Ki-Induced hyperglycaemia and hyperinsulinaemia

**Figure 3:**
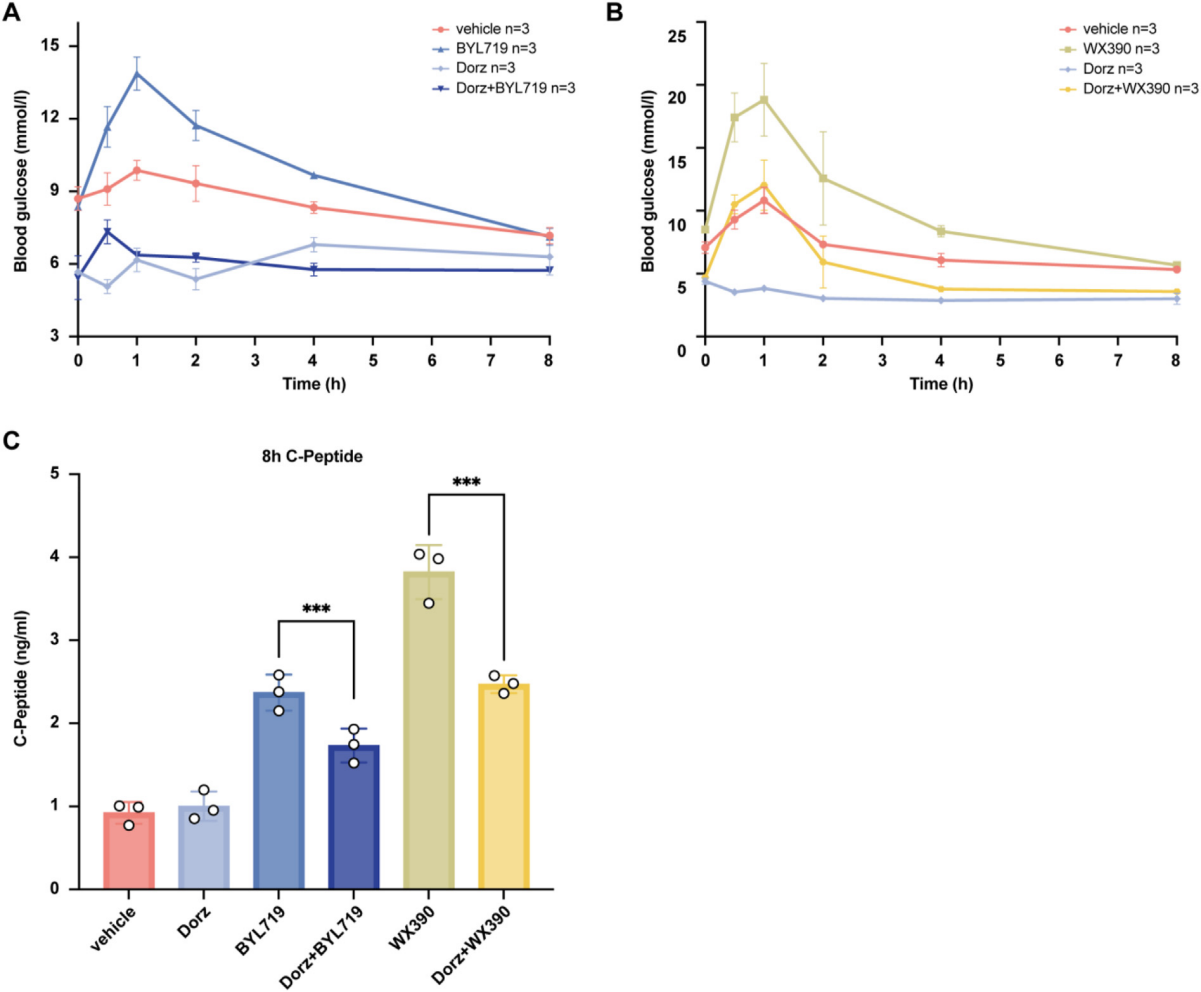
**Dorzagliatin reduces PI3Ki-induced Hyperglycaemia and Hyperinsulinaemia.** Blood glucose changes in (A)BYL719, Dorz, and Dorz + BYL719 group; (B) WX390, Dorz, and Dorz + WX390 group following administration of the indicated treatments. (C) Blood C-peptide levels in BALB/c mice at 8 h after treatment. Data are presented as mean ± SEM; statistical significance was determined by one-way ANOVA: ∗∗∗p < 0.001.

To determine whether dorzagliatin can reduce PI3Ki-induced hyperglycaemia, we used a mouse model of PI3Ki-induced metabolic dysfunction. Mice treated with BYL719 or WX390 alone exhibited significantly elevated blood glucose levels within 1 h of administration. In contrast, co-administration of dorzagliatin with PI3Ki substantial reduced blood glucose levels compared to the PI3Ki-only groups (Figure 3A–B).

Eight hours after dosing, blood C-peptide levels in the dorzagliatin-treated groups were considerably lower than those in the PI3Ki-only groups (Figure 3C). These results indicated that dorzagliatin effectively alleviates PI3Ki-induced hyperglycaemia and hyperinsulinaemia, potentially enhancing the antitumor efficacy of PI3Ki by mitigating the negative effects of elevated insulin levels.

### 3.3 Dorzagliatin enhances the anticancer effects of PI3K inhibitors

**Figure 4:**
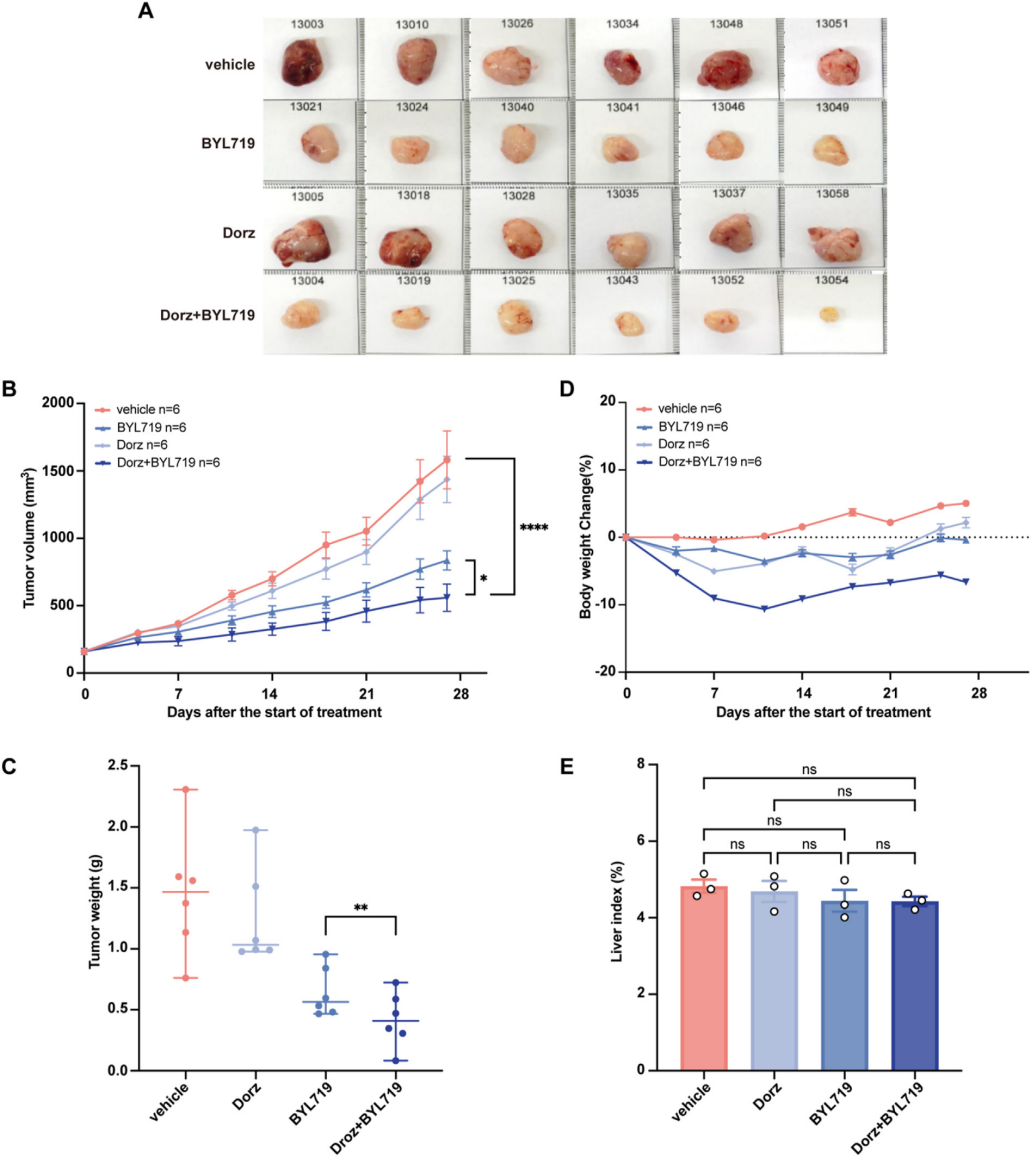
**Dorzagliatin enhances the anticancer effects of PI3Ki.** (A) Images of tumors from the OVCAR3 xenograft model in BALB/c mice after treatment with BYL719 or combination therapy. (B) Tumor growth curves in the OVCAR3 xenograft model following administration of BYL719 or the combination therapy. (C) Tumor weight in different treatment groups. (D) Relative body weight changes (%) in mice during the treatment period. (E) Liver indices calculated at the end of the experiment to assess in vivo toxicity. Data are presented as mean ± SD; statistical significance was determined by one-way ANOVA: ∗p < 0.05, ∗∗p < 0.01, ∗∗∗∗p < 0.0001.

To evaluate whether dorzagliatin enhances the antitumor efficacy of PI3Ki, we compared tumor volumes in mice treated with BYL719 alone versus those receiving the combination therapy. Tumor growth was significantly reduced in the combination therapy group compared to the BYL719-only group (Figure 4A–C), indicating that dorzagliatin and PI3Ki work synergistically to suppress tumors. Relative body weight changes in all treatment groups remained within 15% of baseline, indicating good tolerability of the treatments (Figure 4D). Given the potential toxicity of GKA, as reported in previous studies [26,27], we further evaluated systemic toxicity by calculating the liver index using the formula: Liver Index (%) = (Liver Weight/Mouse Body Weight) × 100%. The results showed that liver indices did not significantly differ between groups, indicating no observable systemic toxicity in major organs (Figure 4E). These findings support the safety and reliability of the combination therapy.

### 3.4 Clinical case study

We report the case of a 52-year-old lady with metastatic cervical cancer who experienced a relapse following a previous platinum-based therapy regimen. She was diagnosed with a squamous cell carcinoma of the cervix with PD-L1 positivity (based on combined positive score) in 2021. After progression of 1st line platinum-based chemotherapy, she was then enrolled onto the phase II trial (NCT06117566) [28] of WX390 (1.1 mg, qd) in combination with Toripalimab (an anti-PD1 antibody, once every 21 days, *iv*) on September 8 2023.

**Figure 5:**
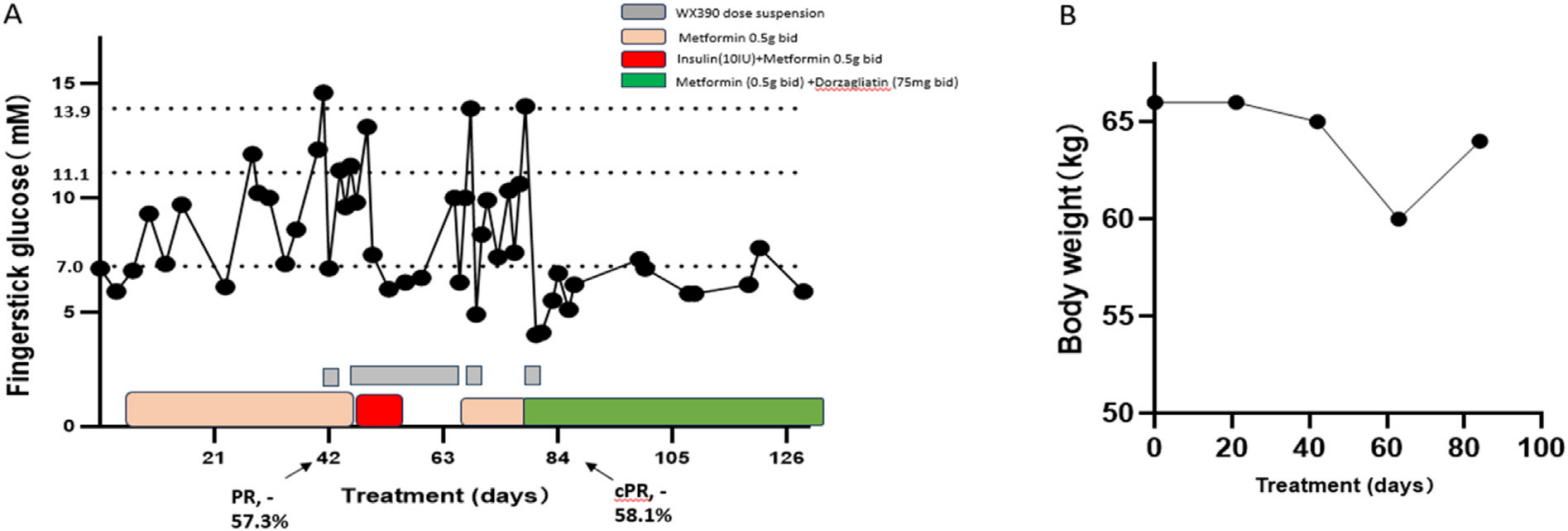
**Blood glucose response to Dorzagliatin in a case of WX390-related hyperglycemia unresponsive to metformin treatment.** (A). Illustrative diagram of the efficacy of Dorzagliatin on a case of WX390-related hyperglycemia unresponsive to metformin treatment. (B). Patient's body weight change during treatment.

Routine fingerstick glucose tests were conducted to monitor the blood glucose levels following WX390 administration. During the first two cycles (21 days per cycle), the patient experienced mild grade 1–2 hyperglycaemia, which was managed effectively with supportive care using metformin (0.5 g, bid). As shown in Figure 5A, her glucose levels were stable during this period. However, at day 41, her blood glucose exceeded 13.9 mM, prompting suspension of WX390 treatment for one day.

A PR (−57.3%) was achieved on day 43 (C3D1) by RECIST v1.1. Despite this, hyperglycaemia became increasingly difficult to control during cycle 3. WX390 dosing was suspended again on day 46, and insulin therapy was initiated from day 50 to day 53 to stabilise her glucose levels. The patient resumed WX390 treatment on day 65, supported by metformin (0.5 g, bid). But monotherapy with metformin failed to adequately control her blood glucose. By day 64 (C4D1), the patient reported fatigue and a significant weight loss, from a baseline weight of 66 kg–60 kg. Dorzagliatin (75 mg, bid) was added to the regimens in combination with metformin from day 75 for supportive care. One week after initiating dorzagliatin, her fingerstick glucose levels stabilised below 7 mM, and her body weight recovered to 64 kg by day 85 (C5D1) (Figure 5B). A confirmed PR was observed at this time, with a tumor size reduction of 58.1% per RECIST v1.1 criteria.

## 4 Discussion

Our results demonstrate that the combination of dorzagliatin with PI3Ki significantly enhances antitumor efficacy compared to PI3Ki alone, as evidenced by the reduced tumor volumes in the experimental models. This therapeutic outcome suggests that the combination therapy utilizes enhanced glycemic control to optimize the effectiveness of PI3Ki, bolstering their ability to suppress tumor growth. The enhanced anticancer efficacy of this combination therapy is likely mediated through multiple signaling pathways, particularly the AKT/INSR/mTOR axis [29]. Notably, the divergent responses to insulin between OVCAR3 and SKOV3 cells in our study may stem from their distinct PI3K/AKT activation mechanisms. In OVCAR3 (PTEN-deficient but PIK3CA wild-type), insulin reactivates AKT via RTK/IRS1 signaling, bypassing PI3K inhibition—a hallmark of PTEN-null tumors reliant on growth factor feedback to evade therapy [COSMIC ID 905933]. Conversely, SKOV3's constitutive PI3K activation (driven by PIK3CA E545K mutation) saturates the pathway, blunting insulin's ability to amplify p-AKT, as seen in PIK3CA-mutant cancers with intrinsic pathway hyperactivity [COSMIC ID 905959]. Dorzagliatin enhances insulin regulation, which prevents hyperactivation of this pathway, allowing PI3Ki to exert more effective suppression of tumor growth. Additionally, by improving the systemic metabolic state, dorzagliatin may diminish metabolic stress on tumor cells, rendering them more vulnerable to the toxic effects of PI3K inhibition. These findings suggest that dorzagliatin holds significant potential as an adjunctive therapy for mitigating PI3Ki-induced hyperglycemia while simultaneously enhancing PI3Ki antitumor efficacy. This dual function could extend the therapeutic window of PI3Ki, enabling higher doses and longer treatment durations with reduced risk of severe hyperglycemia [5].

The Warburg effect, or aerobic glycolysis, is a hallmark metabolic adaptation of cancer cells [30]. This metabolic phenotype supports rapid cancer cell growth by providing not only the precursors for macromolecular biosynthesis but also the energy required for survival [31]. Moreover, it fosters a tumor-supportive microenvironment, conferring a growth advantage to cancer cells [32,33]. Hexokinase, the glycolytic pathway's first and rate-limiting enzyme, catalyzes the phosphorylation of glucose to glucose-6-phosphate [31]. Among the four mammalian isoforms of HK, Type I is predominantly expressed in brain tissues; Type II (HK-2), insulin-sensitive, is mainly found in adipose and muscle tissues; Type III is expressed in trace amounts in liver, kidney, and intestinal tissues; and Type IV, also known as glucokinase, is present in the liver and pancreas. HK-2 is predominantly expressed in insulin-sensitive tissues such as muscle and adipose tissue and is markedly upregulated in rapidly proliferating cancer cells [34]. This upregulation is closely linked to the PI3K/Akt/mTOR signaling pathway, which governs numerous glycolytic enzymes and regulates autophagy[[35], [36], [37]]. Some evidence suggested that the expression of HK-2 changes in various disease states such as cancer. Its activity is closely associated with the Akt/mTORC1 signaling pathway and is regulated by hypoxia-inducible factor HIF-1α (Hypoxia-Inducible Factor-1α) as well as microRNAs such as miR-143 and miR-155. These regulatory mechanisms play significant roles in the development of cancer [38].

In our study, we compared the antitumor efficacy in xenograft tumor tissues between treatment with PI3Ki alone and the combination of dorzagliatin and PI3Ki. Our results demonstrated that the combination therapy significantly suppressed tumor growth. We also observed that elevated insulin levels after PI3Ki alone can be reduced by dorzagliatin, which results in lower blood glucose. These finding indicate that dorzagliatin's ability to regulate glucose metabolism may enhance the antitumor efficacy of PI3Ki by mitigating the metabolic disturbances associated with hyperglycemia. Dorzagliatin achieves this by selectively targeting GK, a key regulator of hepatic glycogen metabolism and pancreatic insulin secretion. GK activity is dynamically modulated by glucose levels and is regulated by interactions with glucokinase regulatory protein (GKRP) in the liver. Under hypoglycemia circumstances, GK forms a complex with GKRP in the nucleus that dissociates when glucose levels rise, allowing GK to catalyze the conversion of glucose to glucose-6-phosphate. This process maintains glucose homeostasis in liver and pancreatic β-cells[[39], [40], [41]]. GK activation supports insulin secretion in response to rising glucose levels, preventing hyperglycemia while avoiding hypoglycemia. This dynamic regulation is particularly important for cancer patients receiving PI3Ki, who require metabolic homeostasis rather than excessive glucose lowering to ensure normal cellular glucose utilization [25]. Dorzagliatin, a novel dual-acting glucokinase activator, simultaneously enhances GK activity in both pancreatic β-cells and hepatic cells, promoting insulin secretion and hepatic glucose utilization. Unlike conventional hypoglycemic agents, dorzagliatin maintains GK activity under hypoglycemic conditions without excessively stimulating insulin secretion, thereby minimizing the risk of hypoglycemia. As a fourth-generation glucokinase activator, dorzagliatin has demonstrated favorable outcomes in phase III clinical trials for type 2 diabetes mellitus [22,23].

The management of PI3Ki-induced hyperglycemia has posed significant challenges in clinical practice. Previous studies, such as the SOLAR-1 [6], BYLieve [42], and Alphabet trials [43], have explored the use of various antidiabetic agents such as metformin, sodium-glucose co-transporter 2 inhibitors (SGLT2i), and insulin to address this problem. In SOLAR-1, among the 187 patients who experienced hyperglycemia of any grade stratified by grouping variable, 163 received pharmacological intervention in the alpelisib cohort. A total of 47 patients (28.8%) required treatment with three or more antihyperglycemic agents. During the study period, 5 out of 12 patients with diabetes mellitus required insulin therapy, and 34 out of 159 patients with prediabetes-initiated insulin use [44]. Metformin, the most commonly used agent, has shown limited efficacy and is often associated with gastrointestinal side effects that overlap with those of PI3Ki, leading to treatment discontinuation [45]. SGLT2i is among the frequently considered therapeutic options for addressing hyperglycemia induced by PI3Ki [46]. Their mechanism of action involves inhibition of SGLT2 in the proximal renal tubules of the kidney and promoting urine glucose excretion by preventing glucose reabsorption [47]. The study findings indicate that comparing patients who received SGLT2i to a matched cohort of patients who did not receive SGLT2i, the incidence of grade ≥3 hyperglycemia adverse events (AE) and hyperglycemia ae leading to alpelisib dose adjustments, interruptions, or discontinuations was reduced by 4.9-fold and 6.4-fold, respectively [48]. SGLT2i also have some side effects, as they excrete excess glucose through the urinary system, which lead to such as ketoacidosis [49], urinary tract infection, and genital infection [50,51]. It is noteworthy that the excretion of glucose as a nutrient may be one of the factors contributing to the acceleration of cachexia progression in cancer patients, thereby impacting the utilization of the medication [52,53]. Insulin, as the most direct and rapid glucose-lowering regimen, has been shown in previous studies and the present study to significantly limit the efficacy of PI3Ki in tumors [44,54]. In contrast, our findings highlight dorzagliatin's unique ability to control PI3Ki-induced hyperglycemia without compromising antitumor efficacy. By simultaneously addressing hyperglycemia and hyperinsulinemia, dorzagliatin preserves the antitumor potential of PI3Ki while providing superior metabolic control. These observations are consistent with emerging evidence supporting the role of GK activation in managing diabetes and its complications, suggesting that dorzagliatin's mechanisms of action may be particularly well-suited to the metabolic disturbances caused by PI3Ki therapy.

Further research is needed to fully understand the molecular mechanisms by which dorzagliatin enhances the efficacy of PI3Ki. Studies could focus on detailed signaling pathway analyses and the role of different metabolic enzymes in tumor suppression. Exploring the potential of dorzagliatin in other cancer types treated with PI3Ki could broaden its clinical application. Investigating the long-term effects of this combination therapy on patient survival and quality of life will also be crucial. Lastly, research into potential biomarkers for predicting patient response to this combination therapy could help personalize treatment plans, maximizing efficacy and minimizing adverse effects.

## 5 Conclusion

Dorzagliatin demonstrates significant potential in addressing PI3Ki-induced hyperglycemia while simultaneously enhancing the antitumor efficacy of PI3Ki therapy. Its ability to selectively activate hepatic GK and modulate insulin levels represents a pivotal mechanism underlying these dual benefits. These findings not only highlight the therapeutic value of dorzagliatin but also underscore the importance of targeting metabolic pathways to augment cancer therapies. Future clinical studies are essential to validate these results in diverse patient populations, while mechanistic investigations will further elucidate the interplay between glucose metabolism and tumor progression. Our studies could establish dorzagliatin as a cornerstone in the management of PI3Ki-related metabolic complications, advancing its translation into routine clinical practice and improving outcomes for patients undergoing PI3Ki therapy.

## CRediT authorship contribution statement

**Guanqin Jin:** Writing – original draft, Visualization, Methodology, Investigation, Data curation, Conceptualization. **Shihuang Liu:** Writing – original draft, Validation, Software, Project administration, Investigation, Funding acquisition, Data curation, Conceptualization. **Kewei Zheng:** Visualization, Validation, Methodology, Investigation, Data curation. **Xiaobo Cheng:** Investigation, Formal analysis. **Ranran Chai:** Investigation, Data curation. **Wei Ye:** Validation, Supervision, Software, Investigation, Data curation. **Wei Wei:** Validation, Supervision, Project administration, Methodology, Investigation, Formal analysis. **Yongguo Li:** Validation, Resources. **Ai Huang:** Resources, Methodology, Investigation, Formal analysis. **Guiling Li:** Writing – review & editing, Validation, Supervision, Methodology, Conceptualization. **Huan Yi:** Writing – review & editing, Visualization, Validation, Resources, Methodology, Investigation, Formal analysis, Data curation, Conceptualization. **Yu Kang:** Writing – review & editing, Validation, Supervision, Resources, Project administration, Funding acquisition, Conceptualization.

## Funding

This work is supported by the 10.13039/501100012166National Key Research and Development Program of China (2022YFC2705004) and Shanghai Key Laboratory of Female Reproductive Endocrine Related Diseases (Obstetrics and Gynecology Hospital, Fudan University) (NO. 20DZ2271300).

## Declaration of competing interest

Wei Ye, Wei Wei and Yongguo Li are employees of Shanghai Jiatan Pharmatech CO. LTD. No other author has reported a potential conflict of interest relevant to this article.

## Data Availability

Data will be made available on request.
